# LNG-IUD and breast cancer: Number 2 – 2026

**DOI:** 10.61622/rbgo/2026FPS2

**Published:** 2026-03-10

**Authors:** Mariane Nunes de Nadai, Carolina Sales de Oliveira, Cristina Falbo Guazzelli, Milena Brito, Zsuzsanna Ilona Katalin de Jármy Di Bella, Ilza Maria Urbano Monteiro

**Affiliations:** Faculdade de Medicina de Bauru Bauru SP Brazil Faculdade de Medicina de Bauru, Bauru, SP, Brazil; Universidade de São Paulo Faculdade de Medicina Ribeirão Preto SP Brazil Faculdade de Medicina, Universidade de São Paulo, Ribeirão Preto, SP, Brazil; Universidade Federal de São Paulo São Paulo SP Brazil Universidade Federal de São Paulo, São Paulo, SP, Brazil; Universidade Federal da Bahia Salvador BA Brazil Universidade Federal da Bahia, Salvador, BA, Brazil; Universidade Federal de São Paulo São Paulo SP Brazil Universidade Federal de São Paulo, São Paulo, SP, Brazil; Universidade Estadual de Campinas Campinas SP Brazil Universidade Estadual de Campinas, Campinas, SP, Brazil

## Key points

- Breast cancer is the most common type of cancer among women worldwide.
- There is concern regarding the use of hormonal contraceptives and their possible relationship with the risk of breast cancer.
- Several studies have evaluated the association between hormonal contraceptives and breast cancer, achieving heterogeneous results.
- Some observational studies and systematic reviews suggest an association between levonorgestrel intrauterine device (LNG-IUD) use and breast cancer. However, these findings are limited by confounding factors and methodological weaknesses in the studies.
- When observed, the increased risk of breast cancer associated with LNG-IUD use is small in absolute terms (occurrence of 1 to 14 additional cases of breast cancer in every 10,000 users of the method) and should be interpreted considering the contraceptive and therapeutic benefits of the method.

## Recommendations

- Breast cancer is a highly prevalent and hormone-dependent disease, which justifies clinical and social concern regarding the use of hormonal contraceptive methods in women of reproductive age.
- Currently available scientific evidence does not consistently and robustly demonstrate a clinically significant increase in the risk of breast cancer associated with LNG-IUD use in the general population.
- The observational studies suggesting a possible association between LNG-IUD and breast cancer present heterogeneous results and have important methodological limitations, especially regarding the inadequate control of confounding factors.
- When identified, the increased risk is small in absolute terms (occurrence of 1 to 14 additional cases of breast cancer in every 10,000 users of the method) and should be interpreted in light of the contraceptive and therapeutic benefits of LNG-IUD use, including high contraceptive efficacy, reduction of uterine bleeding, and protection against endometrial cancer.
- Prescription of the LNG-IUD should be accompanied by evidence-based, clear counseling, considering the patient's clinical, reproductive, and psychosocial context.
- Adopting these recommendations ensures safe clinical practices, prevents unfounded alarmism, and promotes equitable access to effective contraception, thereby fostering a positive impact on both women's reproductive health and society.

## Background

### Why is there concern about the relationship between LNG-IUDs and breast cancer?

The LNG-IUD is a highly effective and reversible long-acting progestogen-only contraceptive (LAPOCs). The concern stems from the fact that breast cancer is hormone-dependent in many cases. Thus, any contraceptive method involving hormones raises questions about a possible impact on the risk of developing and progressing the disease. Some recent observational studies suggesting an association between LNG-IUD use and breast cancer have contributed to broadening this debate among healthcare professionals and patients.

### What is the risk of breast cancer in the general population, regardless of contraceptive use?

Breast cancer is the most common type of cancer among women, representing about 25% of all cases of female cancer.^(1)^ The risk of breast cancer worldwide is approximately 8.3%. In Brazil, this risk is approximately 12% to 13%, meaning that 1 in 8 women will receive a breast cancer diagnosis at some point in their lives.^(1)^ Regardless of exposure to exogenous hormones, several risk factors are associated with the development of the disease, including age, sedentary lifestyle, alcohol use, family history, high breast density, and mutations in genes such as BRCA1 and BRCA2.^(2)^ Given that breast cancer is often hormone-dependent, questions about hormone exposure are pertinent.^(3,4)^ However, the baseline risk of breast cancer exists independently of the use of hormonal contraception.^(5)^

### What do recent studies show about LNG-IUD use and the risk of breast cancer?

Recent systematic reviews have identified conflicting results. While two of them indicated a slight increase in the relative risk of breast cancer associated with LNG-IUD use,^(6,7)^ another review did not demonstrate a statistically significant association.^(8)^ Among the studies cited in these reviews, some showed no evidence of increased risk,^(9–12)^ while others found an association between LNG-IUD use and the development of breast cancer.^(4,11–15)^ One showed this association mainly in patients over 45 years of age^(14)^ and two showed this increase in postmenopausal women.^(4,15,16)^ These differences are mainly related to the methodological heterogeneity of the studies, the variability of the populations analyzed, and the different comparison criteria used. While some studies report an increased risk, the absolute incidence among LNG-IUD users remains very low, as shown in chart 1.

**Chart 1:** Estimated increase in the number of breast cancer cases in users of the levonorgestrel intrauterine device

Article	Risk measure	Estimated increase in cases per 10,000 women
Backman et al. (2005)^(10)^**	Not evaluated	-
Lyytinen et al. (2010)^(15^956^)^*	OR = 1.45	≈ +14
Dinger et al. (2011)^(9)^	OR = 0.99	0
Soini et al. (2014)^(14)^**	SIR = 1.19	≈ 2.9
Heikkinen et al. (2016)^(13)^	OR = 1.32	0
Mørch et al. (2017)^(4)^	RR = 1.21	1.6
Jareid et al. (2018)^(11)^	RR = 1.03	0
Siegelmann et al. (2018)^(12^\" and 2 age-matched non-users as \"controls.\" Exclusion criteria included: prior BC diagnosis, prior (5 years pre-study^)^**	Not evaluated	-
Conz et al. (2020)^(7^especially if used over long periods. Our objective was to conduct a systematic review and meta-analysis of the literature on the risk of breast cancer development in women using the 52-mg levonorgestrel-releasing intrauterine system (LNG-IUS^)^	OR 1.16	≈ 2.0 – 3.0
Silva et al. (2021)^(8)^	OR 1.07	0
Niemeyer et al. (2022)^(16)^	IRR = 1.21	≈ 0.5
Fitzpatrick et al. (2023)^(17)^*	OR 1.32	≈ 4.8
Yi et al. (2024)^(18)^	HR 1.12	1.16
Mørch et al. (2024^)(19^)	HR 1.4	≈ 14
Hadizadeh et al. (2025)^(20)^	RR 1.15–1.25	0.8 – 1.6

RR: relative risk; OR: odds ratio; SIR: standardized incidence ratio

* studies evaluating a population of women over 40 years of age or post-menopausal

** although not calculating RR, authors conclude there is no increased risk

Most of the studies are observational; while they may indicate an association, they do not establish a causal relationship between LNG-IUD use and breast cancer risk. The studies in the meta-analyses^(6–8)^ used varied approaches and, in some instances, failed to fully account for confounding factors. Furthermore, the meta-analyses grouped populations of different ages who used LNG-IUDs for different reasons, such as contraception, treatment of abnormal uterine bleeding, and other medical conditions. The choice of reference populations also varied among the studies: some compared LNG-IUD users with women who had never used hormonal contraceptives, while others used exclusively copper IUD users as a control group. Furthermore, the follow-up time of the studies restricted the findings to short-term associations. Given these limitations, it is not yet possible to precisely define the real relationship between LNG-IUD use and the risk of breast cancer.^(21)^

### How to interpret the increased risk of breast cancer observed in some studies?

There are numerous factors that increase the risk of breast cancer, such as alcohol use (RR 1.1 to 1.5), sedentary lifestyle (RR 1.2 to 1.3), and smoking (RR 1.1 to 1.3).^(5,22)^ It is important to provide guidance on these modifiable risk factors. The increased risk of breast cancer related to LNG IUD use, when identified, is small and corresponds to a minimal impact in absolute terms (an increase of 1 to 14 cases in every 10,000 women). Population estimates indicate that this additional risk is temporary and significantly outweighed by the benefits of effective contraception, including the prevention of unplanned pregnancies and a reduced risk of endometrial and ovarian cancers.^(11,17)^

### Is there biological plausibility for a relevant effect of the LNG IUD on the breast?

Experimental studies demonstrate that levonorgestrel concentrations in the breast tissue of LNG-IUD users are significantly lower than those observed in oral contraceptive users.^(23)^ This finding reduces the biological plausibility of a significant proliferative effect in the breast and weakens the hypothesis of a direct causal relationship.

### What are the main knowledge gaps on this topic?

The LNG-IUD is a highly effective contraceptive widely used across women of all age groups. Beyond contraception, it is indicated for treating gynecological conditions - such as abnormal uterine bleeding, adenomyosis, and dysmenorrhea - and for providing endometrial protection during menopausal hormone therapy. Recently, questions about the relationship between LNG-IUD use and the risk of breast cancer have generated concerns among healthcare professionals and patients. The Brazilian Federation of Gynecology and Obstetrics Associations (Febrasgo) critically reviewed the available scientific evidence in order to offer clear, evidence-based guidelines. Despite the large number of observational studies, high-quality prospective studies with rigorous control of confounding factors and long-term follow-up are still needed, especially in subgroups of women at higher risk for breast cancer.

## Final considerations

A detailed review of the scientific literature, including population cohort studies, case-control studies and systematic reviews with or without meta-analysis, reveals that the results are heterogeneous and, for the most part, do not indicate a significant increase in the risk of breast cancer with LNG-IUD use. Based on the critical analysis of the available evidence, the Febrasgo considers that:

- There is no robust and consistent scientific evidence to support a contraindication to LNG-IUD use based on an increased risk of breast cancer.
- The clinical and non-contraceptive benefits of LNG-IUD use outweigh the potential risks indicated in available observational studies.
- There is no robust and consistent evidence to support a contraindication to LNG-IUD use in women without a diagnosis of breast cancer solely due to the possibility of an increased risk of breast cancer in the general population.
- The possible increased risk of breast cancer observed in some studies is small, heterogeneous, and potentially influenced by biases and confounding factors. The absolute risk is very low (approximately 1 to 14 additional cases of breast cancer per 10,000 LNG-IUD users) and does not outweigh the clinical and therapeutic benefits of LNG-IUD use.
- Prescription should always be accompanied by individualized counseling, based on evidence and the patient's clinical context.

The Febrasgo reaffirms its position that, based on currently available evidence, LNG-IUD use should not be discouraged for fear of an increased risk of breast cancer. This method remains one of the safest, most effective and advantageous options for contraception and treatment of various gynecological conditions.

## Data Availability

The survey data are available in the article.

## National Commission Specialized in contraception of the Brazilian Federation of Gynecology and Obstetrics Associations (Febrasgo)

President:

Ilza Maria Urbano Monteiro

Vice-president:

Rogério Bonassi Machado

Secretary:

Jaqueline Neves Lubianca

Members:

Silvio Silva Fernandes
Carlos Alberto Politano
Cristina Aparecida Falbo Guazzelli
Edson Santos Ferreira Filho
Jarbas Magalhães
Luis Carlos Sakamoto
Mariane Nunes De Nadai
Milena Bastos Brito
Valeria Barbosa Pontes
Carolina Sales Vieira
Zsuzsanna Ilona Katalin De Jarmy Di Bella
Cassia Raquel Teatin Juliato
